# Identification of Candidate Genes Associated with Charcot-Marie-Tooth Disease by Network and Pathway Analysis

**DOI:** 10.1155/2020/1353516

**Published:** 2020-09-23

**Authors:** Min Zhong, Qing Luo, Ting Ye, XiDan Zhu, Xiu Chen, JinBo Liu

**Affiliations:** ^1^Department of Laboratory Medicine, The Affiliated Hospital of Southwest Medical University, 25 Taiping Street, Luzhou, 646000 Sichuan, China; ^2^Department of Neurology, The Affiliated Hospital of Southwest Medical University, 25 Taiping Street, Luzhou, 646000 Sichuan, China

## Abstract

Charcot-Marie-Tooth Disease (CMT) is the most common clinical genetic disease of the peripheral nervous system. Although many studies have focused on elucidating the pathogenesis of CMT, few focuses on achieving a systematic analysis of biology to decode the underlying pathological molecular mechanisms and the mechanism of its disease remains to be elucidated. So our study may provide further useful insights into the molecular mechanisms of CMT based on a systematic bioinformatics analysis. In the current study, by reviewing the literatures deposited in PUBMED, we identified 100 genes genetically related to CMT. Then, the functional features of the CMT-related genes were examined by R software and KOBAS, and the selected biological process crosstalk was visualized with the software Cytoscape. Moreover, CMT specific molecular network analysis was conducted by the Molecular Complex Detection (MCODE) Algorithm. The biological function enrichment analysis suggested that myelin sheath, axon, peripheral nervous system, mitochondrial function, various metabolic processes, and autophagy played important roles in CMT development. Aminoacyl-tRNA biosynthesis, metabolic pathways, and vasopressin-regulated water reabsorption were significantly enriched in the Kyoto Encyclopedia of Genes and Genomes (KEGG) pathway network, suggesting that these pathways may play key roles in CMT occurrence and development. According to the crosstalk, the biological processes could be roughly divided into a correlative module and two separate modules. MCODE clusters showed that in top 3 clusters, 13 of CMT-related genes were included in the network and 30 candidate genes were discovered which might be potentially related to CMT. The study may help to update the new understanding of the pathogenesis of CMT and expand the potential genes of CMT for further exploration.

## 1. Introduction

Charcot-Marie-Tooth Disease (CMT), also known as hereditary motor sensory neuropathy (HMSN), was first reported by French neurologists Charcot and Marie and British neurologist Tooth in 1886 [1, 2]. It is the most common clinical single-gene genetic disease of the peripheral nervous system with high clinical heterogeneity and genetic heterogeneity, with a prevalence of about 1/2500 [3, 4]. Although the disease progresses slowly in most patients, mild to moderate functional impairment does not affect life expectancy. However, there are currently no treatments to reverse the course of CMT, which still limits the ability of patients to move independently, reduces the quality of life, and increases the risk of disability.

Like many other degenerative disorders, hereditary peripheral neuropathies have been difficult to treat. There are currently no effectual pharmacologic treatments for CMT, limiting historic treatment to supportive care. In nearly a decade of research, ascorbic acid, progesterone antagonists, and subcutaneous neurotrophin-3 (NT3) injections have shown initial success in animal models of CMT 1A (the most common subtype of CMT) but have failed to translate any effect in humans [5–8]. Considering the difficulties of treatment, it is imperative to find out the molecular pathogenesis of CMT for the purpose of strategising potential future therapies.

The advent of next-generation sequencing (NGS) has expanded and accelerated the analysis of various diseases at the level of genome, especially in heterogeneous disorder groups such as CMT [9, 10]. Currently, due to the development of NGS, >100 genetic mutations have been found to cause or promote the clinical manifestations of CMT [11, 12]. They have been related to a variety of molecular pathological mechanisms, including either protein synthesis and posttranslational processing (dysfunction of mRNA processing, abnormal endosomal sorting and signaling, aberrant proteasome/protein aggregation, and myelin assembly abnormalities), dysfunction of ion channels (channelopathies), intracellular transportation (axonal transport/cytoskeletal abnormalities), or mitochondrial dysfunction [13, 14]. Based on the understanding of the genetic basis of hereditary neuropathy, different cell and animal models have been established to decode the molecular mechanism of CMT and provide treatment strategies. However, there is no causal treatment for hereditary neuropathy so far. In view of the increasing complexity of the genetics of neuropathies, this paper is aimed at combining bioinformatics with new directions [15].

Although many studies have focused on elucidating the pathogenesis of CMT, few focuses on achieving a systematic analysis of biology to decode the underlying pathological molecular mechanisms. In this study, we firstly made a comprehensive selection of genes genetically related to CMT. We then performed a functional enrichment analysis to identify important biological topics within these genetic factors. In order to further explore the pathogenesis of CMT, we constructed a biological process network to explore possible crosstalk among the significant biological processes. Furthermore, we made a PPI network of these CMT-related genes using MCODE [16, 17]. This study may provide further useful insights into the molecular mechanisms of CMT based on a systematic bioinformatics analysis and find causative mutations in the heterogeneous group of disorders (hereditary neuropathies) in the era of NGS.

## 2. Materials and Methods

### 2.1. Identification of CMT-Related Genes

Candidate genes associated with CMT were collected by retrieving the human genetic association studies deposited in PUBMED (http://www.ncbi.nlm.nih.gov/pubmed/). Referring to published studies [17–19], we searched for reports related to CMT with the term (Charcot-Marie-Tooth Disease [MeSH]) and (polymorphism [MeSH] or genotype [MeSH] or alleles [MeSH]) not (neoplasms [MeSH]). Up to 10 November 2019, a total of 674 publications were retrieved for the disorder. Then, we reviewed the abstracts of all above publications, collecting the genetic association studies of CMT. We narrowed our selection by concentrating on the selected publications reporting a considerable association of one or more genes with CMT. Instead, for the purpose of reducing the number of false-positive finding, we excluded studies reporting negative or insignificant associations, although some genes analyzed in these studies may be associated with CMT. For the selected publications, we reviewed the full texts to assure that the conclusion was consistent with its contents. Thus, we screened out the genes reported to be significantly related to CMT. No ethical approval was required as it did not involve human or animals.

### 2.2. Functional Enrichment Analysis of CMT-Related Genes

The functional features of the CMT-related genes were examined by R software [20, 21] and KOBAS 3.0 [22, 23]. To understand the CMT-related genes underlying biological processes, Gene Ontology analyses (GO; http://geneontology.org) were conducted using R software (version 3.6.2). GO enrichment results were visualized using the R clusterProfiler package (version 3.14.3; 10.18129/B9.bioc.clusterProfiler). GO terms were selected with a false discovery rate (FDR) < 0.05. Kyoto Encyclopedia of Genes and Genomes (KEGG; http://www.genome.jp/kegg) pathway analysis of the candidate genes was performed using the KOBAS online analysis database (available online: http://kobas.cbi.pku.edu.cn/). On the whole, the genes with symbols and/or corresponding NCBI Entrez Gene IDs were uploaded to the server and compared with the genes contained in each canonical pathway according to the KEGG pathway database. All the pathways with one or more genes overlapping with the candidate genes were extracted, and a *P* value was assigned to each pathway through Fisher's exact test to indicate the significance of the overlap between the pathway and the input genes. Then, the pathways with FDR value less than 0.05 were considered to be significantly enriched.

### 2.3. Biological Process Crosstalk Analysis

We further performed biological process crosstalk analysis to explore the interactions among significantly enriched biological processes. To evaluate the overlap between any chosen pairs of biological processes, two measurements were introduced, that is,

(1) $$\begin{array}{c} \text{Jaccard coefficient }\left(\text{JC}\right)=\frac{\left|A\cap B\right|}{\left|A\cup B\right|},\\ \text{Overlap coefficient }\left(\text{OC}\right)=\frac{\left|A\cap B\right|}{\min\left(\left|A\right|,\left|B\right|\right)}, \end{array}$$

where *A* and *B* represent the number of candidate genes in the two biological processes, respectively. To construct the biological process crosstalk, we further implemented the following rules:

1. Select a set of biological processes for crosstalk analysis. Only the biological processes with adjusted *P* values less than 0.05 were used. In the meantime, the biological processes containing less than five candidate genes were removed because biological processes with too few genes may have inadequate biological information
2. Count the number of shared candidate genes between pairs of biological processes, and remove the pair with less than three overlapped genes
3. Calculate the average score of the JC [24] and OC [25] values and rank them
4. Visualize the selected biological process crosstalk with the software Cytoscape (version 3.7.1) [26].

### 2.4. Construction of CMT-Related PPI Network via MCODE

**Figure 1:**
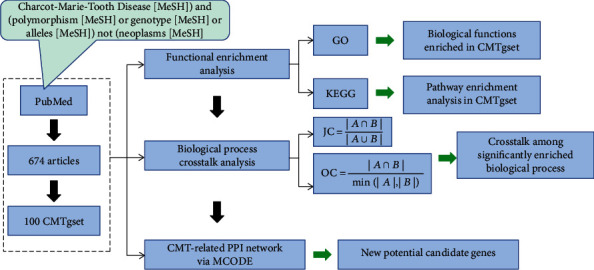
Study design and procedures. The green arrow to the right is the results.

In the current study, we first identified the protein-protein interaction (PPI) network relationships of the CMT-related genes from human Integrated Interaction Database (IID) (available online: http://iid.ophid.utoronto.ca), which is one of the major PPI databases that integrate PPIs from multiple PPI databases, i.e., BioGRID, IntAct, I2D, MINT, InnateDB, DIP, HPRD, BIND, BCI. IID [27, 28] uses PPIs that are computationally predicted by state-of-the-art computational methods [29, 30]. Subsequently, we visualized the PPI network by means of Cytoscape software (version 3.7.1). Furthermore, the Molecular Complex Detection (MCODE) [17, 31, 32] in Cytoscape software was then performed to select appropriate modules of the PPI network to identify the most important MCODE clusters according to clustering scores. The detailed options set as degree cutoff = 2, K-core = 2, node score cutoff = 0.2, and MAX depth = 100. The study design schema is presented in Figure 1.

## 3. Results and Discussion

### 3.1. Identification of Genes Reported to Be Associated with CMT

By searching PUBMED, there were more than 600 studies related to Charcot-Marie-Tooth Disease that were collected. In these publications, it was reported that 100 genes were significantly related to CMT and formed a gene set (CMTgset) for subsequent analysis (Table S1). Among them were seven tRNA synthases: AARS1, GARS1, HARS1, KARS1, MARS1, WARS, and YARS1; four cytochrome c oxidases: COA7, COX10, COX6A1, and SCO2; four heat shock protein family members: DNAJB2, HSPB1, HSPB3, and HSPB8; two dynactins: DCTN1 and DCTN2; and two kinesin family of proteins members: KIF1B and KIF5A. Several genes were those involving the functions associated with fat metabolism (e.g., ABHD12, DGAT2, and MORC2), glucose metabolism (e.g., HK1, NAGLU, and PDK3), mitochondrial-related functional genes (e.g., AIFM1, ATP1A1, DHTKD1, HADHB, MFN2, MPV17, POLG, REEP1, SLC25A46, and SOD1), actin-related genes (e.g., FGD4, FIG4, INF2, MICAL1, and PFN2), and peripheral myelin-related genes (e.g., MPZ, PMP22, and PRX). These data indicated that the genes significantly related to CMT were sophisticated.

### 3.2. Biological Functions Enriched in CMTgset

**Figure 2:**
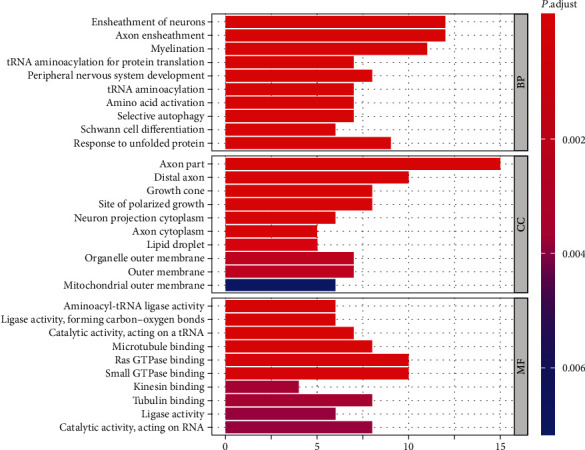
Top 10 significant GO terms and hub gene counts. For each term, the number of enriched genes is indicated by the bar size; while the level of significance is represented by the color. Blue indicates low significance while red represents high significance (FDR < 0.05). GO: Gene Ontology; BP: biological process; CC: cellular component; MF: molecular function; FDR: false discovery rate.

Functional enrichment analysis can show a more specific function of these genes. GO enrichment analysis was performed to investigate the biological function of 100 genes in CMTgset (Table S2). In total, 200 GO terms were significantly enriched in the genes analyzed.  Figure 2 shows the top 10 functions of the three functional assemblies of GO (biological process, cellular component, and molecular function). Among these clusters, some biological processes can be seen: GO terms related to myelin sheath (e.g., response to myelination, response to myelin assembly, and regulation of myelination) and axon (e.g., response to axon ensheathment and response to axonal transport) were enriched in genes in CMTgset. These results were consistent with the clinical classification of CMT, which is generally divided into demyelinating and axonal [33, 34]. Terms directly related to peripheral nervous system (e.g., peripheral nervous system development, myelination in peripheral nervous system, and peripheral nervous system axon ensheathment), Schwann cell (e.g., Schwann cell differentiation and Schwann cell development), mitochondrial function (e.g., mitochondrial fission, mitochondrion organization, and mitochondrial respiratory chain complex IV assembly), and various metabolic processes (e.g., tRNA metabolic process, cellular amino acid metabolic process, nucleoside monophosphate metabolic process, and sphingosine metabolic process) were included. Also, GO terms related to autophagy (e.g., positive regulation of autophagy, process utilizing autophagic mechanism, and regulation of autophagy) were also enriched in these genes. These results revealed that the candidate genes collected were relatively dependable for subsequent bioinformatics analysis.

### 3.3. Pathway Enrichment Analysis in CMTgset

**Figure 3:**
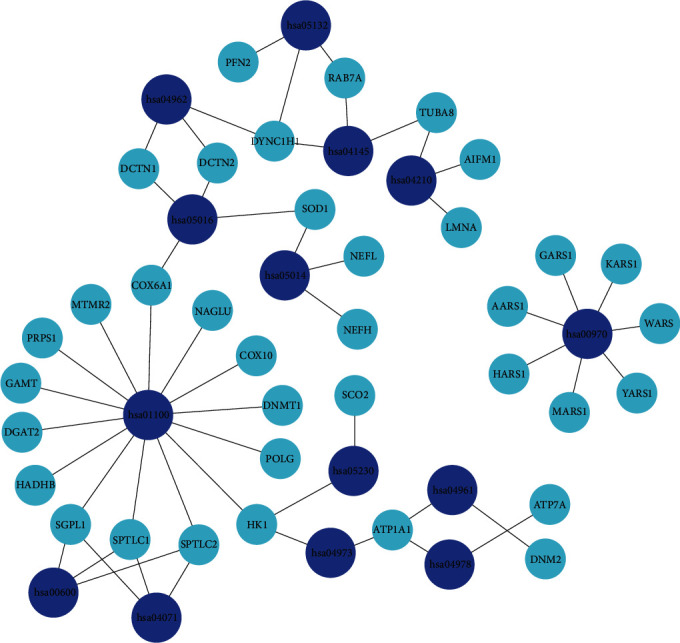
Significant pathway enrichment of CMTgset. Dark blue represents signaling pathway, and light blue represents candidate genes.

Identifying biochemical pathways for the enrichment of candidate genes may provide valuable hints for us to understand the molecular mechanism of CMT. We searched for enriched pathways in the CMTgset using KOBAS 3.0 and found 14 significant enrichment pathways for CMT (Table 1). The signaling pathways of CMTgset were mainly enriched in the aminoacyl-tRNA biosynthesis, metabolic pathways, and vasopressin-regulated water reabsorption. Then use the Cytoscape to calculate the topological characteristics of the network and determine each node. Genes and pathway nodes are represented by semiellipses. The results are shown in Figure 3, in which we can see all the pathways except has00970 (aminoacyl-tRNA biosynthesis) were not independent; instead, they were connected via some genes.

### 3.4. Crosstalk among Significantly Enriched Biological Processes

**Figure 4:**
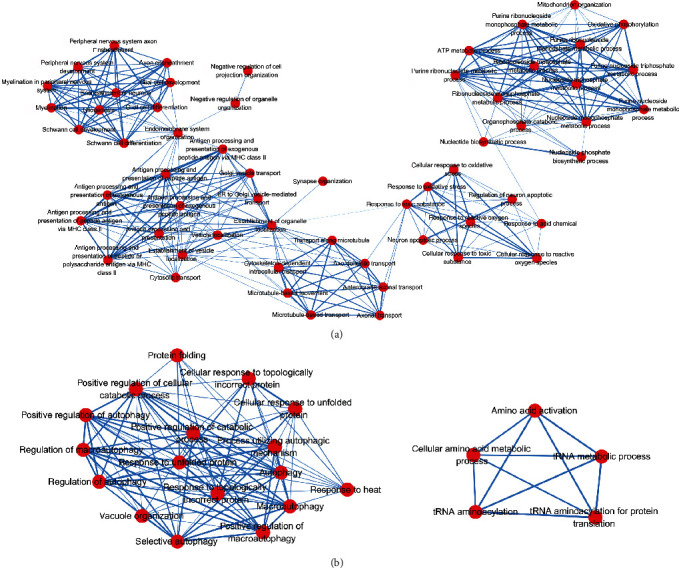
Biological process crosstalk among CMTgset-enriched biological processes. Nodes represent biological processes, and edges represent crosstalk between biological processes. Edge-width corresponds to the score of specific biological process pair. Larger edge-width indicates higher score. (a) Represents one correlative module. (b) Represents two separate modules.

Since CMT might involve many genes and biological processes, taking a further step to understand how significantly enriched biological processes interact with each other, we performed a biological process crosstalk analysis among the 135 significantly enriched biological processes. The method was based on the assumption that two biological processes were considered to crosstalk if they shared a proportion of CMTgset [35]. There were 83 biological processes containing five or more members in CMTgset, of which 81 biological processes met the criterion for crosstalk analysis; i.e., each biological process shared at least three genes with one or more other biological processes. All the biological process pairs (edges) formed by these biological processes were applied to construct the biological process crosstalk, and the overlapping level between two biological processes was measured according to the average scores of coefficients JC and OC. According to their crosstalk, the biological processes could be roughly divided into a correlative module and two separate modules, with each module including biological processes that shared more interactions compared with other biological processes and may likely participate in the same or similar biological processes (Figure 4).

The correlative module also could be grouped into five modules, which could be defined as nervous system-related biological processes (e.g., peripheral nervous system development, ensheathment of neurons, axon ensheathment, and myelination), antigen presentation processes (e.g., antigen processing and presentation of exogenous peptide antigen, antigen processing and presentation of exogenous peptide antigen via MHC class II, and antigen processing and presentation of peptide antigen via MHC class II), transport processes (e.g., transport along microtubule, cytoskeleton-dependent intracellular transport, and axonal transport), oxidation processes (e.g., response to reactive oxygen species, response to oxidative stress, and cellular response to toxic substance), and mitochondrial related biological processes (e.g., mitochondrion organization, purine ribonucleotide metabolic process, and ATP metabolic process). At the same time, these five modules were not independent, but connected by the interaction of several biological processes. The other two separate modules, one related to autophagy (e.g., positive regulation of autophagy and macroautophagy) and one related to tRNA (e.g., tRNA aminoacylation for protein translation, tRNA aminoacylation, and tRNA metabolic process).

### 3.5. Construction of CMT-Specific Protein Network via MCODE

**Figure 5:**
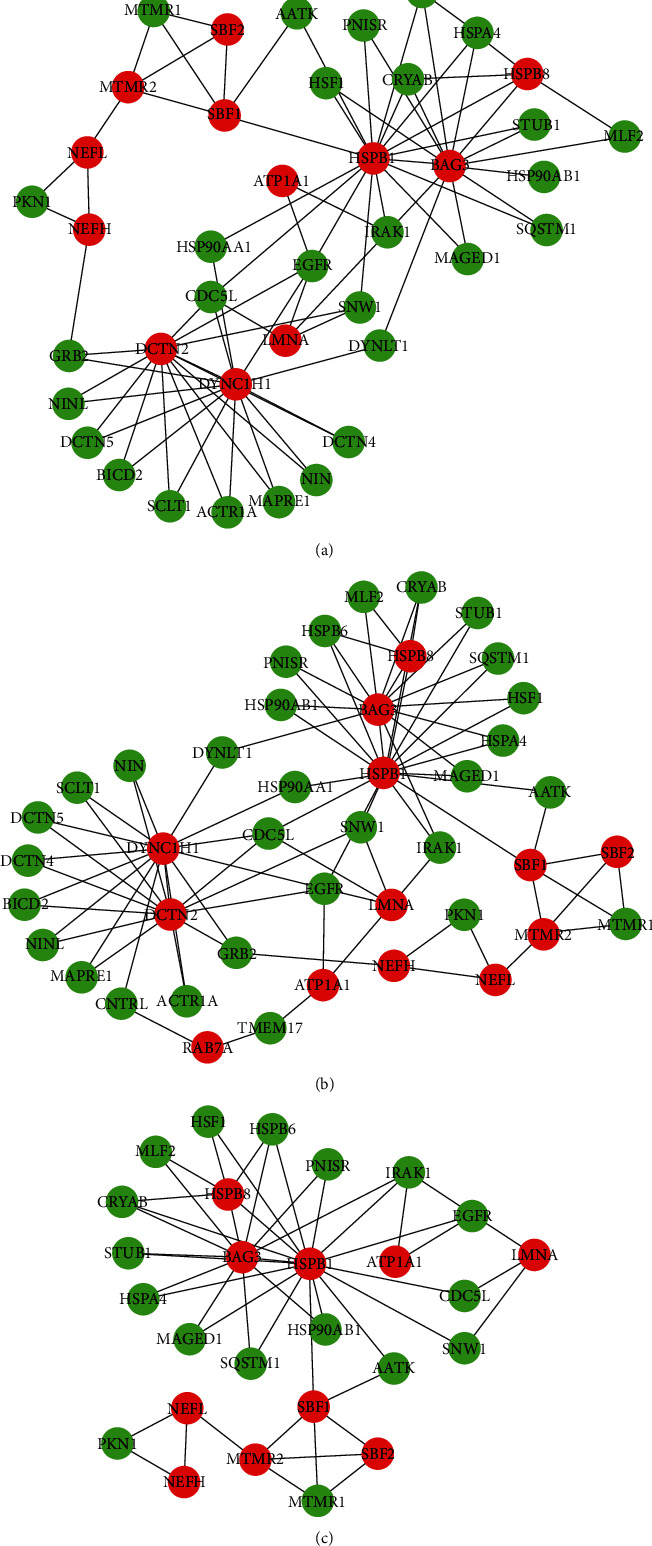
The red nodes are genes of CMTgset, and the green nodes are nonoriginal/extended genes.

Through the online PPI analysis of IID, 88 of the 100 candidate genes can be mapped to the human interactome network, and 1698 predicted genes were screened out. Pathway-based MCODE cluster analysis was used to analyze the topological characteristics of PPI to help understand the potential biological mechanisms associated with the network. The higher the clustering scores, the more important the biological function of this clustering in the development of CMT. Then, we analyzed in detail the three clusters with the highest clustering scores. The results are shown in Figure 5. In Figure 5(a), the PPI cluster (MCODE cluster score = 4) had 40 genes in total, including 12 (30.00%) gene members in the CMTgset, while 28 (70.00%) genes were not included in the list. In Figure 5(b), the PPI cluster (MCODE cluster score = 3.905) had 43 genes in total, including 13 (30.23%) gene members in the CMTgset, but 30 (69.77%) genes were not in the list. In Figure 5(c), the PPI cluster (MCODE cluster score = 3.769) had 27 genes in total, in which 10 (37.04%) gene members were CMTgset but 17 (62.96%) genes have not been reported before. In these 3 PPI clusters, 13 of the CMTgset were included in the human interactome network, among which 30 predicted genes are likely to be highly associated with CMT based on MCODE clusters. These 30 genes are listed in Table 2, which provided a list of new potential candidates for CMT.

## 4. Discussion

In the past few decades, much has been learnt about the molecular mechanisms underlying Charcot-Marie-Tooth Disease from studies on cell models, animals, or human subjects [36–38]. With the high-throughput technology, developing more and more genes/proteins has been recognized to be related to this disorder [15, 39], but it is still far from enough to fully understand the biological processes related to the pathogenesis of CMT at the molecular level. Therefore, the potential pathogenesis of CMT needs to be decoded at the system biology level. In this study, by collecting genes genetically related to CMT and using functional enrichment and network analysis to systematically explore the interaction of these genes, we provided a comprehensive and systematic framework to describe the relevant biochemical processes.

Although genetic association and biochemical studies based on candidate genes have provided us with the knowledge of factors involved in CMT, the systematic approach depicted in this work has clear advantages. Above all, we have comprehensively collected genes potentially genetically related to CMT in our study, which provided a valuable resource for further analysis. From the perspective of molecular network level, it is very important to explore the biological characteristics of genes related to CMT. Moreover, functional enrichment analysis considering the biological relevance of genes can more robustly deal with possible false positives caused by different genes in various studies, and combining with network analysis could provide a more comprehensive view of the molecular mechanism of CMT.

Biological function enrichment analysis revealed the specific biological processes involved by CMTgset. GO enrichment analysis showed that these genes for CMT participated in myelin sheath and axon-related processes, peripheral nervous system, Schwann cell, mitochondrial function, various metabolic processes, and autophagy. In addition, terms such as ensheathment of neurons, axon ensheathment, myelination, tRNA aminoacylation for protein translation, peripheral nervous system development, tRNA aminoacylation, amino acid activation, selective autophagy, Schwann cell differentiation, and response to unfolded protein were in the top ten enriched GO terms, indicating the important roles of these activities in the pathologic processes of CMT. The above findings are basically consistent with previous reports [13, 33, 40, 41].

At the same time, the pathway analysis showed that 14 pathways were enriched and the first of which is aminoacyl-tRNA biosynthesis, which is considered to play an important role in CMT. Aminoacyl-tRNA synthetases (ARSs) are universally expressed enzymes accountable for charging tRNAs with their cognate amino acids, so it is crucial for the first step of protein synthesis [42, 43]. Just as illustrated by a large number of mutations in cytosolic and bifunctional tRNA synthetases causing CMT, the peripheral nerves are often affected [44]. The main mechanisms proposed consist of reduction of aminoacylation activity, alteration of dimerization or localization, interaction of functionally acquired pathogens, and loss of noncanonical functions [45].

Of significance, in biological process crosstalk analysis, we identified a correlative module and two separate modules. The correlative module could be grouped into five modules: the first module was mainly dominated by the biological processes related to the activity of the nervous system. Among these biological processes, axon ensheathment, myelination, peripheral nervous system development, and Schwann cell development have been well studied, involving the axons, myelin sheaths, or peripheral nervous system as well as the progress of Charcot-Marie-Tooth Disease [46–49]. The second module and the third module can be defined together as a transmission function-related biological process. For example, many studies have determined that many major neurodevelopmental and neurodegenerative diseases are associated with mutations in microtubule-associated proteins. The microtubules play the role of tracks for organelle transport, and in neurons, microtubules give assistance to transporting membrane-bound organelles, extending neurites during development, providing scaffolding for neuritis, and maintaining intracellular compartments [13, 50, 51]. Apart from this, insufficient axon transport is a common theme in many neurodegenerative diseases [52, 53], and some researches have found that NEFL [54] and HSPB1 [55] (included in the GO term of axon transport) are closely related to axonal transport dysfunction of CMT. The fourth and fifth modules are both primarily associated with energy-related biological processes, such as oxidative stress response (e.g., response to oxidative stress and oxidative phosphorylation) and mitochondrial related processes (e.g., mitochondrion organization and ATP metabolic process). For the purpose of maintaining membrane excitability and processing neurotransmission and plasticity, neurons rely on mitochondrial function [56]. Moreover, multiple studies have demonstrated that the pathology of CMT has been related to mitochondrial dynamics [57–60]. In addition, this correlative module is connected through several edges, indicating that these biological processes or modules may play a synergistic role in the pathogenesis of CMT, rather than acting alone.

The first three important PPI networks of the CMT-related genes were extracted from the human reference interactome network by MCODE clustering, which extends to its neighboring nodes with the seed node as the center. Then, the nodes that may interact with the seed nodes to build a complex in the PPI network were selected [61, 62]. It is worth noting that 30 extended genes appeared in the first three PPI networks, but were not included in CMT-related genes, which has not been previously reported to be related to CMT. For example, BICD2 (BICD cargo adaptor 2) has been implicated in dynein-mediated, minus end-directed motility along microtubules. Since whole-exome sequencing (WES) has proven to be an efficient tool for mutation screening CMT [10], a novel variant c.1079C>T (p.A360V) in the gene BICD2 is identified in an individual with demyelinating CMT but requires further evidence of pathogenicity [63]. HSP90AA1, HSP90AB1, HSPA4, and HSPB6 are members of the heat shock protein family, which involves many physiological and pathological stresses, such as endoplasmic reticulum stress, protein misfolding and aggregation, and oxidative stress. Most importantly, many members of the heat shock protein family have been shown to resist many degenerative diseases, including CMT [64, 65]. EGFR (epidermal growth factor receptor) is a family member of the ErbB receptor kinase, which can regulate important signaling pathways (including cell proliferation and differentiation, cell cycle, and migration) and stimulate the growth and differentiation of neurons [66, 67]. There is increasing evidence that ErbB receptor-mediated signaling plays an important role in controlling Schwann cell axon communication and myelination in the peripheral nervous system [68]. Genes play a vital biological role in the form of proteins. So, the above events or functions reflected by the PPI network may be closer to the actual biological processes of CMT-related genes. As demonstrated by the results elaborated above, our methods extend the findings of previous studies and provide more information about the pathogenesis of CMT. At the same time, our MCODE cluster analysis not only provided a meaningful inferred network of CMT-related genes but also has the potential to identify potential candidate genes.

So far, the detection and analysis approaches of CMT are diverse, including linkage analysis, functional studies, and studies in the model organisms. However, the research of this disorder's molecular mechanism have not yet broken through, which may be due to the inability of conventional single-gene analyses to explain complicated psychiatric phenotype. This paper takes a comprehensive analysis of potential causal genes within a pathway [35, 69] and/or a network [70, 71] framework which might provide more important insights. In addition, the data set CMTgset obtained by the approach proposed in this paper included the most recent researches. In CMTgset, we used a unified gene symbol standard to collect genes that are significantly related to CMT reported by the original authors. Since most of the genes came from association researches on individual genes, some of the genes may only have a moderate *P* value, but they worked synergistically with other genes to show a significant association with CMT, which made CMTgset a more comprehensive data set for CMT exploration.

Of course, this study still has a few limitations. First of all, our functional enrichment analysis and PPI network analysis results are entirely dependent on the genes reported in the retrieved literature that are related to CMT, but more evidence needs to be used to further verify specific new genes. In other words, the results obtained by/in the bioinformatics approach should be further verified in the DNA samples obtained from CMT-affected patients. Secondly, our research accepts the results of the original authors of each retrieved study. Due to the imbalance and incompleteness of these existing studies, they will certainly bias our results. Third, to reduce the false positive rate of genes, we excluded publications that were negative or irrelevant. However, some of the genes in these studies may be related to the pathogenesis of CMT and were only excluded because of the small sample size or heterogeneity or other factors. In addition, although the quantity and quality of PPI databases have been greatly improved recently, the human interaction group is still incomplete and has many false positives.

## 5. Conclusion

In this study, we analyzed related genes collected from selected literature deposited in PUBMED, using the system biology framework to conduct a comprehensive, systematic biological function and network-based analysis of CMT. By integrating the information from GO, pathways, and biological process crosstalk analysis, the conclusions of this study may help to update the new understanding of the pathogenesis of CMT and expand the potential genes of CMT for further exploration.

## Figures and Tables

**Table 1:** Pathways enriched in CMTgset.

Pathways	ID	*P* value^a^	*P*_BH_ value^b^	Genes included in the pathway^c^
Aminoacyl-tRNA biosynthesis	hsa00970	7.19*E* − 10	2.09*E* − 08	GARS1, WARS, AARS1, HARS1, MARS1, YARS1, KARS1
Metabolic pathways	hsa01100	3.16*E* − 06	4.25*E* − 05	NAGLU, GAMT, SGPL1, COX10, COX6A1, MTMR2, PRPS1, DNMT1, SPTLC2, HADHB, HK1, DGAT2, SPTLC1, POLG
Vasopressin-regulated water reabsorption	hsa04962	2.29*E* − 04	1.63*E* − 03	DCTN1, DCTN2, DYNC1H1
Sphingolipid metabolism	hsa00600	2.76*E* − 04	1.89*E* − 03	SGPL1, SPTLC2, SPTLC1
Amyotrophic lateral sclerosis (ALS)	hsa05014	3.46*E* − 04	2.29*E* − 03	SOD1, NEFL, NEFH
Salmonella infection	hsa05132	1.49*E* − 03	7.65*E* − 03	PFN2, RAB7A, DYNC1H1
Huntington's disease	hsa05016	1.57*E* − 03	7.98*E* − 03	DCTN1, COX6A1, SOD1, DCTN2
Sphingolipid signaling pathway	hsa04071	3.81*E* − 03	1.61*E* − 02	SGPL1, SPTLC2, SPTLC1
Apoptosis	hsa04210	5.67*E* − 03	2.21*E* − 02	LMNA, TUBA8, AIFM1
Carbohydrate digestion and absorption	hsa04973	6.52*E* − 03	2.48*E* − 02	HK1, ATP1A1
Endocrine and other factor-regulated calcium reabsorption	hsa04961	6.79*E* − 03	2.55*E* − 02	ATP1A1, DNM2
Phagosome	hsa04145	7.46*E* − 03	2.75*E* − 02	TUBA8, RAB7A, DYNC1H1
Mineral absorption	hsa04978	8.19*E* − 03	2.95*E* − 02	ATP1A1, ATP7A
Central carbon metabolism in cancer	hsa05230	1.31*E* − 02	4.30*E* − 02	HK1, SCO2

CMTgset: Charcot-Marie-Tooth Disease-related gene set. ^a^*P* values were calculated by Fisher's exact test. ^b^*P*_BH_ values were adjusted by the Benjamini and Hochberg (BH) method. ^c^One hundred CMT-related genes included in the pathway.

**Table 2:** Genes included in CMT top three specific PPI networks but not in the CMTgset.

Gene symbol	Gene name	Cluster
MTMR1	Myotubularin-related protein 1	Clusters A, B, and C
AATK	Apoptosis-associated tyrosine kinase	Clusters A, B, and C
HSF1	Heat shock transcription factor 1	Clusters A, B, and C
PNISR	PNN interacting serine and arginine-rich protein	Clusters A, B, and C
HSPB6	Heat shock protein family B (small) member 6	Clusters A, B, and C
CRYAB	Crystallin alpha B	Clusters A, B, and C
HSPA4	Heat shock protein family A (Hsp70) member 4	Clusters A, B, and C
STUB1	STIP1 homology and U-box containing protein 1	Clusters A, B, and C
MLF2	Myeloid leukemia factor 2	Clusters A, B, and C
HSP90AB1	Heat shock protein 90 alpha family class B member 1	Clusters A, B, and C
SQSTM1	Sequestosome 1	Clusters A, B, and C
MAGED1	MAGE family member D1	Clusters A, B, and C
IRAK1	Interleukin 1 receptor-associated kinase 1	Clusters A, B, and C
EGFR	Epidermal growth factor receptor	Clusters A, B, and C
SNW1	SNW domain containing 1	Clusters A, B, and C
CDC5L	Cell division cycle 5 like	Clusters A, B, and C
PKN1	Protein kinase N1	Clusters A, B, and C
DYNLT1	Dynein light chain Tctex-type 1	Clusters A and B
GRB2	Growth factor receptor bound protein 2	Clusters A and B
NINL	Ninein like	Clusters A and B
DCTN5	Dynactin subunit 5	Clusters A and B
BICD2	BICD cargo adaptor 2	Clusters A and B
SCLT1	Sodium channel and clathrin linker 1	Clusters A and B
ACTR1A	Actin-related protein 1A	Clusters A and B
MAPRE1	Microtubule-associated protein RP/EB family member 1	Clusters A and B
NIN	Ninein	Clusters A and B
DCTN4	Dynactin subunit 4	Clusters A and B
HSP90AA1	Heat shock protein 90 alpha family class A member 1	Clusters A and B
CNTRL	Centriolin	Cluster B
TMEM17	Transmembrane protein 17	Cluster B

CMT = Charcot-Marie-Tooth Disease; PPI = protein-protein interaction.

## Data Availability

The statistical data of the article used to support the findings of this study are available from the corresponding author upon request.

## Supplementary Materials

Supplementary Materials Supplemental Table 1: list of genes associated with Charcot-Marie-Tooth Disease. Supplemental Table 2: Gene Ontology and biological process terms enriched in CMT-related genes.
