# Supplementary material for: Identification of Candidate Genes Associated with Charcot-Marie-Tooth Disease by Network and Pathway Analysis
Source: Biomed Res Int. 2020 Sep 23;2020:1353516. doi: 10.1155/2020/1353516 (PMC7532371; doi:10.1155/2020/1353516)
Supplement: Supplementary Materials — Supplemental Table 1: list of genes associated with Charcot-Marie-Tooth Disease. Supplemental Table 2: Gene Ontology and biological process terms enriched in CMT-related genes. [file 1353516.f1.doc]

**Supplemental table 1** List of genes associated with Charcot-Marie-Tooth Disease

| **Gene symbol** | **Gene name** | **Reference^*^** |
| --- | --- | --- |
| AARS1 | alanyl-tRNA synthetase 1 | 30124830; 28902413; 26032230; 22573628; 22009580 |
| ABHD12 | abhydrolase domain containing 12, lysophospholipase | 31393079 |
| AIFM1 | apoptosis inducing factor mitochondria associated 1 | 25110935 |
| ARHGEF10 | Rho guanine nucleotide exchange factor 10 | 26143528; 25025039 |
| ATP1A1 | ATPase Na+/K+ transporting subunit alpha 1 | 29499166 |
| ATP7A | ATPase copper transporting alpha | 21143467 |
| BAG3 | BLC2-associated athanogene 3 | 30145633; 28754666 |
| BSCL2 | BSCL2 lipid droplet biogenesis associated, seipin | 23553728 |
| C1ORF194 | chromosome 1 open reading frame 194 | 31199454 |
| CNTNAP1 | contactin associated protein 1 | 31397905 |
| COA7 | cytochrome c oxidase assembly factor 7 (putative) | 29718187 |
| COX10 | cytochrome c oxidase assembly factor heme A | 11381029 |
| COX6A1 | cytochrome c oxidase subunit 6A1 | 25152455 |
| CTDP1 | RNA polymerase II subunit A C-terminal domain phosphatase | 29174527 |
| DCTN1 | dynactin subunit 1 | 12627231 |
| DCTN2 | dynactin subunit 2 | 26517670 |
| DGAT2 | diacylglycerol O-acyltransferase 2 | 26786738 |
| DHTKD1 | dehydrogenase E1 and transketolase domain containing 1 | 30896807; 28902413; 25110935 |
| DNAJB2 | DnaJ heat shock protein family (Hsp40) member B2 | 26752306; 25274842 |
| DNM2 | dynamin 2 | 29473246; 24102355; 22091729; 18394888; 17825552 |
| DNMT1 | DNA methyltransferase 1 | 21532572 |
| DRP2 | dystrophin related protein 2 | 26227883 |
| DYNC1H1 | dynein cytoplasmic 1 heavy chain 1 | 21820100 |
| EGR2 | early growth response 2 | 29174527; 28902413; 22522483; 17717711; 15947997; 10915613; 10762521; 10502832 |
| FBLN5 | fibulin 5 | 23945280; 23328402; 21576112 |
| FGD4 | FYVE, RhoGEF and PH domain containing 4 | 28902413; 28847448; 26400421 |
| FGF13 | fibroblast growth factor 13 | 27438001 |
| FIG4 | FIG4 phosphoinositide 5-phosphatase | 29742619; 28902413; 24878229; 23489662; 21705420; 21655088; 18353139 |
| GAMT | guanidinoacetate N-methyltransferase | 29449460 |
| GAN | gigaxonin | 27852232; 26492578 |
| GARS1 | glycyl-tRNA synthetase 1 | 28355569; 22144914; 20169446; 17142907; 17101916 |
| GDAP1 | ganglioside induced differentiation associated protein 1 | 31179332; 30692068; 29896895; 29694336; 29396836; 28902413; 28395795; 28379183; 28244113; 26848201; 26362287; 25231362; 23456260; 22546700; 22200116; 21753178; 21212451; 19922348; 19500985; 19381883; 18991200; 18504680; 18492089; 18421898; 17433678; 15377708; 15192818; 14561495; 12566285; 11743580; 11743579 |
| GJB1 | gap junction protein beta 1 | 30896807; 29710024; 29236290; 29174527; 28902413; 28469099; 28283593; 27804109; 27098783; 27088055; 26454100; 25850958; 25595958; 24819634; 24724718; 23912496; 23384994; 21104867; 20730878; 20532933; 20472869; 20193560; 20128140; 19062535; 17646144; 17159110; 17052905; 15947997; 12775342; 12715686; 12207932; 11446387; 11295246; 11266688; 11140841; 11030070; 11024208; 10873293; 10587015; 10586279; 10220155; 10071100;  9760211; 9600589; 9566397; 9541114; 9469571; 9328258; 9401007; 9018031; 8990008; 8790370; 8698335; 8628473; 8889588; 8829637; 7615296; 9856562 |
| GNB4 | G protein subunit beta 4 | 28642160; 25110935; 23434117 |
| HADHB | hydroxyacyl-CoA dehydrogenase trifunctional multienzyme complex subunit beta | 24314034 |
| HARS1 | histidyl-tRNA synthetase 1 | 25110935 |
| HINT1 | histidine triad nucleotide binding protein 1 | 28902413; 25342199; 25231362; 25110935 |
| HK1 | hexokinase 1 | 28902413; 26822750; 23996628 |
| HSPB1 | heat shock protein family B (small) member 1 | 29330367; 28902413; 28828227; 28379183; 20660910; 18832141; 18587268; 16215937; 16087758 |
| HSPB3 | heat shock protein family B (small) member 3 | 29341343 |
| HSPB8 | heat shock protein family B (small) member 8 | 20225027 |
| IGHMBP2 | immunoglobulin mu DNA binding protein 2 | 28902413; 28202949; 26298607; 25439726 |
| INF2 | inverted formin 2 | 31515790; 28902413; 27088055; 23945280; 22187985 |
| JPH1 | junctophilin 1 | 30804591; 25168384 |
| KARS1 | lysyl-tRNA synthetase 1 | 20920668 |
| KIF1B | kinesin family member 1B | 15136675; 12884740 |
| KIF5A | kinesin family member 5A | 28902413 |
| LITAF | lipopolysaccharide induced TNF factor | 27088055; 25342198; 23576546; 23359569; 20709679; 15776429 |
| LMNA | lamin A/C | 28902413; 27405450; 20709679; 18549403; 12467734; 11799477 |
| LRSAM1 | leucine rich repeat and sterile alpha motif containing 1 | 28902413; 22781092; 20865121 |
| MARS1 | methionyl-tRNA synthetase 1 | 28148924; 25110935 |
| MCM3AP | minichromosome maintenance complex component 3 associated protein | 28633435 |
| MED25 | mediator complex subunit 25 | 30039206; 23781959; 19290556 |
| MFN2 | mitofusin 2 | 31315766; 30649465; 29898954; 29752145; 29361379; 29174527; 28902413; 28660751; 28076385; 27907123; 27088055; 26955893; 26916081; 26801520; 26686600; 26454100; 26382835; 25850958; 24863639; 24819634; 24803844; 24126688; 23840650; 22926664; 22762946; 22546700; 22526351; 21508331; 21258814; 20350294; 20163430; 20008656; 19427854; 18946002; 17940179; 17444508; 17437620; 16762064; 16714318; 16437557; 16043786; 15549395 |
| MICAL1 | microtubule associated monooxygenase, calponin and LIM domain containing 1 | 26752306 |
| MID2 | midline 2 | 25110935 |
| MME | membrane metalloendopeptidase | 28855494; 27588448 |
| MORC2 | MORC family CW-type zinc finger 2 | 26659848 |
| MPV17 | mitochondrial inner membrane protein MPV17 | 26437932 |
| MPZ | myelin protein zero | 31315766; 29896895; 29174527; 28902413; 27088055; 26454100; 26310628; 26234237; 25850958; 25720167; 25025039; 24819634; 23811036; 23564290; 22622165; 22275255; 22222859; 21787890; 21503568; 20937820; 20556410; 20456450; 19629567; 18422810; 18209201; 17940173; 17030746; 16543539; 15377707; 15261887; 15094849; 15050444; 14638973; 12911457; 12845552; 12207932; 10764043; 10586254; 10545037; 10533074; 10071056; 921723; 88167085; 8956034; 8844219; 8664899; 7550231; 7527371; 7509228; 7693129 |
| MTMR2 | myotubularin related protein 2 | 28509084; 23781959; 20709679; 12398840; 11354824 |
| NAGLU | N-acetyl-alpha-glucosaminidase | 25818867 |
| NDRG1 | N-myc downstream regulated 1 | 29724652; 29174527; 28902413; 28776325; 24136616; 24028195; 23996628; 15922294; 12872253 |
| NEFH | neurofilament heavy | 29587262 |
| NEFL | neurofilament light | 31315766; 29191368; 28902413; 28501821; 27088055; 26645395; 19286384; 18758688; 17620486; 17052987; 12673592; 10848490 |
| PDK3 | pyruvate dehydrogenase kinase 3 | 28902413; 26801680; 25110935; 23297365 |
| PFN2 | profilin 2 | 29449460 |
| PLEKHG5 | pleckstrin homology and RhoGEF domain containing G5 | 23844677 |
| PMP22 | peripheral myelin protein 22 | 31315766; 29896895; 29771329; 29729827; 29691480; 29573232; 29174527; 28902413; 28374912; 28108290; 27577214; 27288457; 27088055; 27009151; 26921370; 26454100; 26378787; 25850958; 25648254; 25522693; 25500726; 25385046; 25025039; 24819634; 24646194; 22530759; 22131320; 20842290; 20416338; 19930872; 19909487; 19888301; 19705173; 19067730; 18698610; 15205993; 15099590; 14502374; 12885335; 11112660; 11058901; 11017934; 10586256; 10330345; 10078969; 9678704; 9554482; 9409359;  9005872; 8630243; 8956034; 8752424; 1341967; 9335255 |
| PNKP | polynucleotide kinase 3'-phosphatase | 30039206 |
| POLG | DNA polymerase gamma, catalytic subunit | 28902413; 25025039; 18195151 |
| PRPS1 | phosphoribosyl pyrophosphate synthetase 1 | 31434166 |
| PRX | periaxin | 28902413; 23781959; 18504680; 16770524; 15197604 |
| PTRH2 | peptidyl-tRNA hydrolase 2 | 28328138 |
| RAB7A | RAB7A, member RAS oncogene family | 26362287; 20709679; 17060578 |
| REEP1 | receptor accessory protein 1 | 29124833; 28902413 |
| SBF1 | SET binding factor 1 | 30039846; 28902413; 25110935; 23749797 |
| SBF2 | SET binding factor 2 | 30028002; 29896895; 28902413; 25462154; 15304601 |
| SCN9A | sodium voltage-gated channel alpha subunit 9 | 28902413 |
| SCO2 | synthesis of cytochrome C oxidase 2 | 29351582 |
| SETX | senataxin | 25025039 |
| SGPL1 | sphingosine-1-phosphate lyase 1 | 28077491 |
| SH3TC2 | SH3 domain and tetratricopeptide repeats 2 | 31227790; 29321516; 28902413; 28355569; 27882734; 26829735; 25231362; 24833716; 21291453; 20220177; 19805030; 19272779; 18846676; 18511281; 17470135; 16924012; 16806930 |
| SIGMAR1 | sigma non-opioid intracellular receptor 1 | 31167812 |
| SLC12A6 | solute carrier family 12 member 6 | 26752306 |
| SLC25A46 | solute carrier family 25 member 46 | 26168012 |
| SOD1 | superoxide dismutase 1 | 28579206; 25025039; 22475618 |
| SOX10 | SRY-box transcription factor 10 | 24833716 |
| SPG11 | SPG11 vesicle trafficking associated, spatacsin | 26556829 |
| SPTLC1 | serine palmitoyltransferase long chain base subunit 1 | 11242114 |
| SPTLC2 | serine palmitoyltransferase long chain base subunit 2 | 28902413 |
| SURF1 | SURF1 cytochrome c oxidase assembly factor | 24027061 |
| TFG | trafficking from ER to golgi regulator | 27653917; 25110935 |
| TRIM2 | tripartite motif containing 2 | 25893792; 25110935 |
| TRPV4 | transient receptor potential cation channel subfamily V member 4 | 28902413; 26362287; 20037587; 12682323 |
| TTR | transthyretin | 28902413; 27212199 |
| TUBA8 | tubulin alpha 8 | 26752306 |
| VCP | valosin containing protein | 25125609 |
| WARS | tryptophanyl-tRNA synthetase | 31069783 |
| YARS1 | tyrosyl-tRNA synthetase 1 | 17545306; 16429158 |

^*^ References are designated by PMIDs, which are the identifiers of PubMed. PMIDs in each gene term are equal and there’re not priorities in the permutation of the

corresponding PMIDs of specified genes.

**Supplemental table 2** Gene Ontology Biological Process terms enriched in CMT-related genes

| **GO Biological Process Terms^a^** | | **No. of genes^b^** | | **P-value^c^** | | **P_BH_-value^d^** | |
| --- | --- | --- | --- | --- | --- | --- | --- |
| GO:0007272 | ensheathment of neurons | | 12 | | 3.70E-12 | | 4.09E-09 |
| GO:0008366 | axon ensheathment | | 12 | | 3.70E-12 | | 4.09E-09 |
| GO:0042552 | myelination | | 11 | | 5.62E-11 | | 4.14E-08 |
| GO:0006418 | tRNA aminoacylation for protein translation | | 7 | | 2.53E-09 | | 1.36E-06 |
| GO:0007422 | peripheral nervous system development | | 8 | | 3.51E-09 | | 1.36E-06 |
| GO:0043039 | tRNA aminoacylation | | 7 | | 4.20E-09 | | 1.36E-06 |
| GO:0043038 | amino acid activation | | 7 | | 4.94E-09 | | 1.36E-06 |
| GO:0061912 | selective autophagy | | 7 | | 4.94E-09 | | 1.36E-06 |
| GO:0014037 | Schwann cell differentiation | | 6 | | 4.38E-08 | | 1.07E-05 |
| GO:0006986 | response to unfolded protein | | 9 | | 2.22E-07 | | 4.64E-05 |
| GO:0022011 | myelination in peripheral nervous system | | 5 | | 2.52E-07 | | 4.64E-05 |
| GO:0032292 | peripheral nervous system axon ensheathment | | 5 | | 2.52E-07 | | 4.64E-05 |
| GO:0014044 | Schwann cell development | | 5 | | 4.50E-07 | | 7.12E-05 |
| GO:0035966 | response to topologically incorrect protein | | 9 | | 4.51E-07 | | 7.12E-05 |
| GO:0008088 | axo-dendritic transport | | 6 | | 9.42E-07 | | 0.0001 |
| GO:0010970 | transport along microtubule | | 8 | | 1.08E-06 | | 0.0001 |
| GO:0099111 | microtubule-based transport | | 8 | | 1.08E-06 | | 0.0001 |
| GO:0008090 | retrograde axonal transport | | 4 | | 1.40E-06 | | 0.0002 |
| GO:0016239 | positive regulation of macroautophagy | | 6 | | 1.51E-06 | | 0.0002 |
| GO:0030705 | cytoskeleton-dependent intracellular transport | | 8 | | 1.88E-06 | | 0.0002 |
| GO:0010508 | positive regulation of autophagy | | 7 | | 2.65E-06 | | 0.0003 |
| GO:0034614 | cellular response to reactive oxygen species | | 8 | | 3.15E-06 | | 0.0003 |
| GO:0008089 | anterograde axonal transport | | 5 | | 3.43E-06 | | 0.0003 |
| GO:0007033 | vacuole organization | | 7 | | 7.08E-06 | | 0.0007 |
| GO:0098930 | axonal transport | | 5 | | 8.09E-06 | | 0.0007 |
| GO:0019886 | antigen processing and presentation of exogenous peptide antigen via MHC class II | | 6 | | 1.43E-05 | | 0.0012 |
| GO:0002495 | antigen processing and presentation of peptide antigen via MHC class II | | 6 | | 1.71E-05 | | 0.0014 |
| GO:0002504 | antigen processing and presentation of peptide or polysaccharide antigen via MHC class II | | 6 | | 1.81E-05 | | 0.0014 |
| GO:0000266 | mitochondrial fission | | 4 | | 2.27E-05 | | 0.0017 |
| GO:0006399 | tRNA metabolic process | | 7 | | 2.61E-05 | | 0.0019 |
| GO:0007018 | microtubule-based movement | | 8 | | 2.93E-05 | | 0.0021 |
| GO:0051656 | establishment of organelle localization | | 11 | | 3.05E-05 | | 0.0021 |
| GO:0021782 | glial cell development | | 6 | | 3.09E-05 | | 0.0021 |
| GO:0034599 | cellular response to oxidative stress | | 9 | | 3.26E-05 | | 0.0021 |
| GO:0000302 | response to reactive oxygen species | | 8 | | 3.54E-05 | | 0.0022 |
| GO:0007005 | mitochondrion organization | | 11 | | 4.36E-05 | | 0.0027 |
| GO:0006914 | autophagy | | 11 | | 4.80E-05 | | 0.0028 |
| GO:0061919 | process utilizing autophagic mechanism | | 11 | | 4.80E-05 | | 0.0028 |
| GO:0009896 | positive regulation of catabolic process | | 10 | | 5.20E-05 | | 0.0029 |
| GO:0098840 | protein transport along microtubule | | 3 | | 6.68E-05 | | 0.0036 |
| GO:0099118 | microtubule-based protein transport | | 3 | | 6.68E-05 | | 0.0036 |
| GO:0043217 | myelin maintenance | | 3 | | 8.19E-05 | | 0.0043 |
| GO:0031331 | positive regulation of cellular catabolic process | | 9 | | 9.70E-05 | | 0.0050 |
| GO:0006520 | cellular amino acid metabolic process | | 9 | | 0.0001 | | 0.0056 |
| GO:0016236 | macroautophagy | | 8 | | 0.0001 | | 0.0056 |
| GO:0009123 | nucleoside monophosphate metabolic process | | 9 | | 0.0001 | | 0.0056 |
| GO:0032288 | myelin assembly | | 3 | | 0.0001 | | 0.0056 |
| GO:0006979 | response to oxidative stress | | 10 | | 0.0001 | | 0.0063 |
| GO:0008535 | respiratory chain complex IV assembly | | 3 | | 0.0001 | | 0.0063 |
| GO:0010001 | glial cell differentiation | | 7 | | 0.0002 | | 0.0068 |
| GO:0019430 | removal of superoxide radicals | | 3 | | 0.0002 | | 0.0095 |
| GO:0070841 | inclusion body assembly | | 3 | | 0.0003 | | 0.0107 |
| GO:0016241 | regulation of macroautophagy | | 6 | | 0.0003 | | 0.0113 |
| GO:0021680 | cerebellar Purkinje cell layer development | | 3 | | 0.0003 | | 0.0113 |
| GO:0071450 | cellular response to oxygen radical | | 3 | | 0.0003 | | 0.0113 |
| GO:0071451 | cellular response to superoxide | | 3 | | 0.0003 | | 0.0113 |
| GO:0010506 | regulation of autophagy | | 8 | | 0.0003 | | 0.0118 |
| GO:0097237 | cellular response to toxic substance | | 7 | | 0.0003 | | 0.0128 |
| GO:0017004 | cytochrome complex assembly | | 3 | | 0.0004 | | 0.0134 |
| GO:0002478 | antigen processing and presentation of exogenous peptide antigen | | 6 | | 0.0004 | | 0.0134 |
| GO:0009636 | response to toxic substance | | 10 | | 0.0004 | | 0.0134 |
| GO:0009150 | purine ribonucleotide metabolic process | | 10 | | 0.0004 | | 0.0134 |
| GO:0019884 | antigen processing and presentation of exogenous antigen | | 6 | | 0.0004 | | 0.0137 |
| GO:0000303 | response to superoxide | | 3 | | 0.0004 | | 0.0141 |
| GO:0009161 | ribonucleoside monophosphate metabolic process | | 8 | | 0.0004 | | 0.0147 |
| GO:0051650 | establishment of vesicle localization | | 7 | | 0.0004 | | 0.0151 |
| GO:0000305 | response to oxygen radical | | 3 | | 0.0005 | | 0.0151 |
| GO:0009060 | aerobic respiration | | 4 | | 0.0005 | | 0.0164 |
| GO:0034620 | cellular response to unfolded protein | | 5 | | 0.0005 | | 0.0174 |
| GO:0048002 | antigen processing and presentation of peptide antigen | | 6 | | 0.0006 | | 0.0174 |
| GO:0000045 | autophagosome assembly | | 4 | | 0.0006 | | 0.0175 |
| GO:0007626 | locomotory behavior | | 6 | | 0.0007 | | 0.0195 |
| GO:0072384 | organelle transport along microtubule | | 4 | | 0.0007 | | 0.0195 |
| GO:1905037 | autophagosome organization | | 4 | | 0.0007 | | 0.0195 |
| GO:0051648 | vesicle localization | | 7 | | 0.0007 | | 0.0195 |
| GO:0006119 | oxidative phosphorylation | | 5 | | 0.0007 | | 0.0212 |
| GO:0009165 | nucleotide biosynthetic process | | 8 | | 0.0008 | | 0.0216 |
| GO:0010256 | endomembrane system organization | | 8 | | 0.0008 | | 0.0230 |
| GO:1901293 | nucleoside phosphate biosynthetic process | | 8 | | 0.0008 | | 0.0230 |
| GO:0035967 | cellular response to topologically incorrect protein | | 5 | | 0.0008 | | 0.0231 |
| GO:0042063 | gliogenesis | | 7 | | 0.0009 | | 0.0248 |
| GO:0046034 | ATP metabolic process | | 7 | | 0.0009 | | 0.0255 |
| GO:0016482 | cytosolic transport | | 5 | | 0.0010 | | 0.0259 |
| GO:0043523 | regulation of neuron apoptotic process | | 6 | | 0.0010 | | 0.0259 |
| GO:0019882 | antigen processing and presentation | | 6 | | 0.0013 | | 0.0315 |
| GO:0006878 | cellular copper ion homeostasis | | 2 | | 0.0013 | | 0.0315 |
| GO:0006971 | hypotonic response | | 2 | | 0.0013 | | 0.0315 |
| GO:0046512 | sphingosine biosynthetic process | | 2 | | 0.0013 | | 0.0315 |
| GO:0090309 | positive regulation of methylation-dependent chromatin silencing | | 2 | | 0.0013 | | 0.0315 |
| GO:0048193 | Golgi vesicle transport | | 7 | | 0.0013 | | 0.0324 |
| GO:0050808 | synapse organization | | 8 | | 0.0013 | | 0.0326 |
| GO:0046434 | organophosphate catabolic process | | 6 | | 0.0014 | | 0.0329 |
| GO:0031641 | regulation of myelination | | 3 | | 0.0014 | | 0.0335 |
| GO:0007009 | plasma membrane organization | | 4 | | 0.0015 | | 0.0335 |
| GO:0043473 | pigmentation | | 4 | | 0.0015 | | 0.0335 |
| GO:0045833 | negative regulation of lipid metabolic process | | 4 | | 0.0015 | | 0.0335 |
| GO:0045104 | intermediate filament cytoskeleton organization | | 3 | | 0.0015 | | 0.0335 |
| GO:0051646 | mitochondrion localization | | 3 | | 0.0015 | | 0.0335 |
| GO:0001845 | phagolysosome assembly | | 2 | | 0.0015 | | 0.0335 |
| GO:0046520 | sphingoid biosynthetic process | | 2 | | 0.0015 | | 0.0335 |
| GO:0099188 | postsynaptic cytoskeleton organization | | 2 | | 0.0015 | | 0.0335 |
| GO:1905383 | protein localization to presynapse | | 2 | | 0.0015 | | 0.0335 |
| GO:0009205 | purine ribonucleoside triphosphate metabolic process | | 7 | | 0.0016 | | 0.0341 |
| GO:0045103 | intermediate filament-based process | | 3 | | 0.0016 | | 0.0345 |
| GO:0009167 | purine ribonucleoside monophosphate metabolic process | | 7 | | 0.0017 | | 0.0349 |
| GO:0009408 | response to heat | | 5 | | 0.0017 | | 0.0349 |
| GO:0048284 | organelle fusion | | 4 | | 0.0017 | | 0.0349 |
| GO:0009126 | purine nucleoside monophosphate metabolic process | | 7 | | 0.0017 | | 0.0349 |
| GO:0009199 | ribonucleoside triphosphate metabolic process | | 7 | | 0.0017 | | 0.0352 |
| GO:0009144 | purine nucleoside triphosphate metabolic process | | 7 | | 0.0018 | | 0.0358 |
| GO:0006670 | sphingosine metabolic process | | 2 | | 0.0018 | | 0.0358 |
| GO:0032252 | secretory granule localization | | 2 | | 0.0018 | | 0.0358 |
| GO:0071472 | cellular response to salt stress | | 2 | | 0.0018 | | 0.0358 |
| GO:0099640 | axo-dendritic protein transport | | 2 | | 0.0018 | | 0.0358 |
| GO:0051402 | neuron apoptotic process | | 6 | | 0.0019 | | 0.0358 |
| GO:0031334 | positive regulation of protein complex assembly | | 6 | | 0.0019 | | 0.0363 |
| GO:0021695 | cerebellar cortex development | | 3 | | 0.0020 | | 0.0373 |
| GO:0031345 | negative regulation of cell projection organization | | 5 | | 0.0021 | | 0.0391 |
| GO:0001101 | response to acid chemical | | 7 | | 0.0021 | | 0.0391 |
| GO:0055070 | copper ion homeostasis | | 2 | | 0.0022 | | 0.0398 |
| GO:0090308 | regulation of methylation-dependent chromatin silencing | | 2 | | 0.0022 | | 0.0398 |
| GO:0009141 | nucleoside triphosphate metabolic process | | 7 | | 0.0024 | | 0.0429 |
| GO:0006888 | ER to Golgi vesicle-mediated transport | | 5 | | 0.0024 | | 0.0438 |
| GO:0006684 | sphingomyelin metabolic process | | 2 | | 0.0025 | | 0.0444 |
| GO:0006825 | copper ion transport | | 2 | | 0.0025 | | 0.0444 |
| GO:0031937 | positive regulation of chromatin silencing | | 2 | | 0.0025 | | 0.0444 |
| GO:0032781 | positive regulation of ATPase activity | | 3 | | 0.0027 | | 0.0455 |
| GO:0050885 | neuromuscular process controlling balance | | 3 | | 0.0027 | | 0.0455 |
| GO:0097366 | response to bronchodilator | | 3 | | 0.0027 | | 0.0455 |
| GO:0006457 | protein folding | | 5 | | 0.0027 | | 0.0457 |
| GO:0050905 | neuromuscular process | | 4 | | 0.0027 | | 0.0459 |
| GO:0010759 | positive regulation of macrophage chemotaxis | | 2 | | 0.0029 | | 0.0480 |
| GO:0033617 | mitochondrial respiratory chain complex IV assembly | | 2 | | 0.0029 | | 0.0480 |
| GO:0046519 | sphingoid metabolic process | | 2 | | 0.0029 | | 0.0480 |
| GO:0010639 | negative regulation of organelle organization | | 7 | | 0.0030 | | 0.0494 |

^a^ Only the leaf GO Biological Process terms containing 2 or more CMT-related genes are shown.

^b^ Number of genes in the 100 CMT-related genes and also in the category

^c^ P-values were calculated by hypergeometric test

^d^ P_BH_-values were adjusted by Benjamini & Hochberg (BH) method
